# Effect of the Presence of Antibiotic Residues on the Microbiological Quality and Antimicrobial Resistance in Fresh Goat Meat

**DOI:** 10.3390/foods11193030

**Published:** 2022-09-30

**Authors:** Jessica Da Silva-Guedes, Alba Martinez-Laorden, Elena Gonzalez-Fandos

**Affiliations:** Department of Food Technology, CIVA Research Center, University of La Rioja, Madre de Dios, 26006 Logroño, Spain

**Keywords:** food safety, small ruminants, antibiotic residues, enterococci, staphylococci, Methicillin-resistant *S. aureus* (MRSA), *Macrococcus* spp.

## Abstract

A total of 11 fresh goat legs were collected at the retail level. Mesophiles, *Pseudomonas* spp., *Enterobacteriaceae*, *staphylococci*, *enterococci*, *Clostridium perfringens, Campylobacter* spp., and *Listeria monocytogenes* counts were determined. Nine samples were free of antibiotic residues, while in the other two samples the presence of sulfadiazine and doxycycline was detected. The antimicrobial resistance of *E. coli*, *staphylococci*, *Macrococcus* spp., and *enterococci* isolates was also evaluated. *Clostridium perfringens* was found in two samples. Methicillin-resistant *Staphylococcus aureus* was detected in one sample. *S. epidermidis* isolated from one sample containing doxycycline residues showed resistance to mupirocin. Moreover, multi-resistant *S. epidermidis* and *M. caseolyticus* were found. Most of the isolated *Enterococcus faecium* were multi-resistant. Neither extended-spectrum β-lactamase -producing *E. coli* nor vancomycin-resistant enterococci were detected in any sample. The presence of doxycycline or sulfadiazine could affect the goat meat microbiota since less microbial diversity was found in these samples compared to those free of antibiotics. The presence of antibiotic residues could increase the antimicrobial resistance of *enterococci* in fresh goat meat. The presence of multidrug-resistant bacteria in goat meat could be considered a potential threat and should be monitored. Special measures should be taken at the farm level and during slaughter to reduce antimicrobial resistance.

## 1. Introduction

Goat meat is very popular in many regions, including the Middle East, Africa, Mediterranean, Caribbean, and Southeast Asia [1]. Spain has a large goat population, mainly used to produce milk, although goat meat production is also important [2]. In the last two decades an increasing demand for goat meat has been observed in other areas, mainly due to the habits of some ethnic groups and the popularity of ethnic dishes [3]. Fresh goat meat is usually sold in small pieces, the legs being the most widely marketed and consumed part.

Several studies have pointed out that fresh meat, including goat meat, is a rich medium for microbial growth [4]. The microflora present in fresh meat is very heterogeneous and comprises *Pseudomonas* spp., *Brochotrix thermosphacta*, *Lactobacillus* spp. *Enterobacteriaceae,* and *Acinetobacter* spp. [4,5,6]. These bacteria can produce meat spoilage depending on their levels and species [4]. *Pseudomonas* spp. is considered the most prevalent bacteria in fresh meat stored under aerobic conditions [7], while lactic acid bacteria (LAB) and *B. thermosphacta* are the main bacteria involved in the spoilage of fresh meat packaged under modified atmospheres, although these bacteria can also be found in fresh meat stored under aerobic conditions [8,9]. Moreover, pathogens can be present in meat. The most important foodborne bacterial pathogens associated with meat are *Salmonella* spp., *Escherichia coli*, *Staphylococcus aureus*, *Listeria monocytogenes*, *Campylobacter jejuni*, *Clostridium perfringens,* and *Yersinia enterocolitica*. [4,10]. Pathogens associated with meat from small ruminants (goat and sheep) include mainly *Clostridium perfringens*, *C. jejuni*. *E. coli* O157:H7, *Listeria monocytogenes,* and *Salmonella* spp. [11].

Most of the studies on fresh goat meat have been carried out in Asian and African countries [1,10,12]. Few works are available on the microbiological quality of goat meat in developed countries [2,13]. Most of the investigations have been focused on mesophiles, coliforms, *E. coli*, *Salmonella*, *Enterobacteriaceae,* and *Pseudomonas* spp. [1,10]. Less information is available on *Clostridium* spp., *Listeria monocytogenes,* and *Enterococcus* [12,13]. Most of the studies carried out in developing countries have pointed out that goat meat was highly contaminated due to the hygienic and sanitary conditions of meat processing and inadequate storage temperatures [1,10]. It should be noted that the main source of microbiological contamination of carcasses along the slaughter line is of fecal origin, and consequently, *Enterobacteriaceae* and *E*. *coli* are considered useful indicators of hygienic conditions of the slaughtering, further processing, and handling [14,15].

Nowadays, the increase in antimicrobial resistance is considered a great threat to animal and human health [16]. The spread of extended-spectrum β-lactamase (ESBL)-producing *Enterobacteriaceae* is of special concern [17]. Several studies have pointed out that *E. coli* isolated from goat meat has a significant level of antimicrobial resistance [18]. Moreover, the presence of extended-spectrum β-lactamase (ESBL)-producing *E. coli* has been reported in goat meat [19], being considered a great concern in food safety. Methicillin-resistant *S. aureus* (MRSA) is responsible for a large number of human hospital-acquired infections [20]. It should be noted that MRSA has been isolated from goat meat [21]. *Enterococci* are part of the natural intestinal microbiota of animals and can be found in goat meat [2]. The ability of *enterococci* to acquire resistance to antibiotics and their possible role as a reservoir of antibiotic resistance genes that could be transferred to other bacteria is of great concern, especially the resistance to vancomycin [22].

The use of antibiotics can promote the presence of antibiotic-resistant strains in animals, and it can lead to being released into humans through the consumption of meat. Therefore, there is special concern about the role of food of animal origin in the spread of antimicrobial resistance [17].

In a previous study, the presence of antibiotic residues was analyzed in 5357 commercialized meat samples from the Spain-France cross-border area (POCTEFA region), comprising territories in both Spain and France [23]. Meat samples from 12 different species were collected from five different cities: Logroño (Spain), Zaragoza (Spain), Bilbao (Spain), Perpignan (France), and Toulouse (France) [23]. In that previous study, only samples of goat meat were taken in Logroño (Spain), and 18.2% of them showed the presence of antibiotics at levels above those established by the European Union Regulations [23,24]. It should be noted that in that previous study only 5 meat samples of 5357 showed antibiotic residues above the maximum residue limits (MRLs) (2 from goat, 1 from lamb, 1 from rabbit, and 1 from pork) [23]. These previous results encouraged us to study the microbiota in goat meat and the effect of antibiotic residues.

The aim of this study was to evaluate the effect of the presence of antibiotic residues on the microbiological quality and safety of fresh goat meat, besides the effect on antimicrobial resistance.

## 2. Materials and Methods

### 2.1. Meat Samples and Microbiological Analysis

A total of eleven fresh goat meat samples were collected from different retailers in Logroño (Spain). All the samples purchased were legs, since it is the most frequently marketed part. Samples were transported to the laboratory under refrigeration and analyzed as soon as possible. The samples were obtained as in a previous study in which the presence of antibiotic residues was analyzed in 5357 commercialized meat samples [23]. The retailers selected for sample purchase were representative of the different trade models, and the number of samples of each species depended on consumption data, availability, and diversity of commercial brands, which was particularly low in goat meat [23]. The consumption of goat meat is linked to regional dietary habits, and its consumption is low compared to other kinds of meat such as beef or pork [23]. The 11 meat samples were evaluated by the UPLC-QTOF method (Ultra Performance Liquid Chromatography-Quadrupole Time of Flight) for the detection of antibiotic residues, as shown in a previous work [23]. Doxycycline and sulfadiazine were found in two samples at levels of 813 µg/kg and 164.3 µg/kg, respectively. Antibiotic residues were not detected in the other nine samples [23]. The levels detected in the positive samples exceeded the maximum residue limits (MRLs) of antimicrobials in meat established by Regulation 37/2010 (100 µg/kg) [24].

For microbiological analysis, ten grams of each meat sample were aseptically weighed and homogenized with 90 mL of 0.1% sterile peptone water (Oxoid, Basingstoke, Hampshire, UK) in a Masticator blender (IUL Instruments, Barcelona, Spain) for 2 min. Serial dilutions were prepared using the same diluent [25]. The following microbiological analyses were performed: mesophiles, *Pseudomonas* spp., *Enterobacteriaceae*, *staphylococci*, *enterococci*, *Clostridium perfringens*, *Listeria* spp, and *Campylobacter* spp. Mesophile counts were determined on Plate Count Agar (Scharlau, Barcelona, Spain) and incubated at 30 °C for 48 h [25]. *Pseudomonas* spp were determined on a chromogenic agar for *Pseudomonas* (Scharlau, Barcelona, Spain) incubated at 30 °C for 72 h [25]. *Enterobacteriaceae* counts were determined on MacConkey agar (Oxoid, Basingstoke, Hampshire, UK)) incubated at 37 °C for 24 h [26]. *Staphylococci* were determined on Mannitol Salt Agar (Oxoid, Basingstoke, Hampshire, UK)) incubated at 35 °C for 36 h [26]. *Enterococci* were determined in Kanamycin Esculin Azide agar (Scharlau, Barcelona, Spain) incubated at 37 °C for 48 h [2]. *Clostridium perfringens* were determined on Tryptose Sulphite Cycloserine agar (Merck, Darmstadt, Germany) incubated at 40 °C for 24 h under anaerobic conditions [12]. *Campylobacter* counts were determined on Brilliance CampyCount agar (Oxoid, Basingstoke, Hampshire, UK)) incubated at 42 °C for 48 h under microaerobic conditions using the Campygen kit (Oxoid, Basingstoke, Hampshire, UK)) [27]. *Listeria monocytoegenes* counts were determined on Agar *Listeria* according to Ottaviani and Agosti (ALOA agar) (BioMérieux, Marcy l’Etoile, France) incubated at 30 °C for 24 h [27]. The presence of *Campylobacter* spp. and *L. monocytogenes* was determined as previously described [27].

Also, a screening for the determination of ESBL-producing *E. coli*, methicillin-resistant *S. aureus*, and vancomycin-resistant enterococci (VRE) was carried out [17]. Two grams of meat were taken and incubated in flasks containing 50.0 mL of Brain Heart Infusion (BHI) broth (Oxoid, Basingstoke, Hampshire, UK) at 37 °C for 24 h. After incubation, the samples were plated with the streak plate method on the following chromogenic media: CHROMID ESBL^®^ agar, CHROMID MRSA^®^ agar, and CHROMID VRE^®^ agar (BioMérieux, Marcy l’Etoile, France). Presumptive ESBL-producing *E. coli*, MRSA, and vancomycin-resistant *enterococci* colonies were selected for further analysis.

### 2.2. Isolation and Identification

From each sample and culture media five colonies of the highest dilution that yielded growth were randomly selected and isolated. The morphology of suspected colonies was taken into account when specific media were used. Isolates were purified on Tryptone Soy Agar (Scharlau, Barcelona, Spain) and Brain Heart Infusion broth (Scharlau, Barcelona, Spain). The purified isolates were kept at −80 °C. Bacterial identification was performed by a Matrix-Assisted Laser Desorption/Ionization-Time of Flight Mass-Spectrometry (MALDI-TOF MS) Biotyper (Bruker, Billerica, MA, USA).

### 2.3. Phenotypic Confirmation of ESBL Producers

Phenotypic confirmation of ESBL producers was carried out using the double disc method using cefotaxime (30 µg), ceftazidime (30 µg), and amoxycillin/clavulanic acid (20 + 10 µg) (Oxoid, Basingstoke, Hampshire, UK)) [28]. This test was also applied to all the *E. coli* isolates.

### 2.4. Methicillin Resistance of S. aureus

The methicillin resistance of *S. aureus* was performed according to the Clinical Laboratory Standards Institute guidelines [29], by the diffusion agar assay using cefoxitin (30 μg).

### 2.5. Resistance of Staphylococci and Macrococcus spp. Isolates

The antimicrobial susceptibility of 18 staphylococci and 6 *Macrococcus caseolyticus* isolates was tested against a panel of 29 antimicrobials using the disk diffusion method on Mueller–Hinton agar. For each specie identified, one strain was selected for each different media and sample. The following antibiotic disks (Oxoid, Basingstoke, Hampshire, UK) were used: amikacin (AK, 30 µg), cefoxitin (FOX, 30 µg), ceftaroline (CPT, 30 µg), chloramphfenicol (C, 30 µg), ciprofloxacin (CIP, 5 µg), clindamycin (CMN, 2 µg), doxycycline (DO, 30 µg), fusidic acid (FAD, 10 µg), enrofloxacin (ENR, 5 µg), erythromycin (ERY, 15 µg), gatifloxacin (GAT, 5 µg), gentamicin (CN, 10 µg), kanamycin (K, 30 µg), levofloxacin (LEV, 5 µg), lincomycine (MY, 15 µg), linezolid (LZD, 30 µg), minocycline (MH, 30 µg), mupirocin (PUM, 200 µg), nitrofurantoin (F, 300 µg), norfloxacin (NOR, 5 µg), penicillin (P, 10 UI), rifampicin (RD, 5 µg), streptomycin (S, 10 UI), sulfadiazine (SUZ, 300 µg), trimethoprim -sulfamethoxazole (SXT 1.25:23.75 µg), tedizolid (TZD, 2 µg), tetracycline (TE, 30 µg), tobramycin (TOB, 10 µg), trimethoprim (W, 5 µg), tylosin (TY, 30 µg), and vancomycin (VA, 30 µg). For *S. saprophyticus*, quinupristin-dalfopristin (QD, 15 µg) was also tested, and in the case of *S. aureus* also benzylpenicillin (PNG, 1 UI). After incubation at 37 °C for 18 to 24 h, inhibition zones were measured and scored as susceptible, intermediate (reduced susceptibility), or resistant according to the Clinical and Laboratory Standards Institute guidelines [20]. For *M. caseolyticus*, the resistance breakpoints for *Staphylococcus* spp. were used as suggested by Cotting et al. [30].

### 2.6. Resistance of Enterococci Isolates

The antimicrobial susceptibility of 14 *enterococci* isolates was tested against a panel of 16 antimicrobials using the disk diffusion method on Mueller–Hinton agar. For each specie identified one strain was selected for each different media and sample. The following antibiotic disks (Oxoid, Basingstoke, Hampshire, UK) were used: ampicillin (AMP, 10 µg), chloramphenicol (C, 30 µg), ciprofloxacin (CIP, 5 µg), doxycycline (DO, 30 µg), enrofloxacin (ENR, 5 µg), gentamicin (CN, 120 µg), imipenem (IMP, 5 µg), levofloxacin (LEV, 5 µg), linezolid (LZD, 30 µg), minocycline (MH, 30 µg), nitrofurantoin (F, 300 µg), norfloxacin (NOR, 10 µg), teicoplanin (TEC, 30 µg), tetracycline (TE, 30 µg), tigecycline (TGC, 15 µg), and vancomycin (VA, 30 µg). After incubation at 37 °C for 18 to 24 h, inhibition zones were measured and scored as susceptible, intermediate (reduced susceptibility), or resistant according to the Clinical and Laboratory Standards Institute guidelines [29].

The minimum inhibitory concentration (MIC) for tetracycline, enrofloxacin, and ciprofloxacin was assessed by E-test strips (Biomerieux^®^ Marcy l’Etoile, France) in those enterococci isolates that showed resistance or reduced susceptibility to these antibiotics.

### 2.7. Statistical Analysis

Analysis of variance was carried out using SPSS version 26 software (IBM SPSS Statistics) [31]. Tukey’s test for comparison of means was performed using the same program. The level of significance was determined at *p* < 0.05.

## 3. Results

Mesophile counts in samples with antibiotic residues (P) and free of antibiotic residues (N) were 5.31 ± 1.49 and 4.72 ± 1.36 log CFU/g, respectively. No significant differences (*p* > 0.05) in mesophile counts were found between the samples with antibiotic residues and those free of antibiotics. Mesophile counts in the sample with doxycycline at levels of 813 µg/kg showed significantly (*p* < 0.05) lower counts (3.36 ±0.02 log CFU/g) than the sample with sulfadiazine at levels of 164.3 µg/kg (6.08 ± 0.03 log CFU/g).

The bacteria identified from Plate Count Agar in samples with the presence of antibiotic residues or free of antibiotic residues are shown in Table 1. The microbial groups identified in samples with the presence of residues were lactic acid bacteria (58.33%), *B. thermosphacta* (16.67%), and other Gram-negative bacteria that included *Microbacterium* spp. and *Psychrobacter* spp. (25%). The predominant bacteria in samples free of antibiotic residues were *B. thermosphacta* (35.85%), followed by *Pseudomonas* spp. (26.42%). Moreover, *Micrococcaceae* (13.21%), *Enterobacteriaceae* (5.66%), *enterococci* (3.77%), and other Gram-negative bacteria (15.09%) (*Acinetobacter* spp., *Chryseobacterium scophtalnum*, *Brevundimonas diminuta*, and *Sphingobacterium faecium*) were isolated from these antibiotic-free samples.

*Pseudomonas* spp. counts were below the detection limit (<1 log CFU/g) in samples with antibiotic residues. *Pseudomonas* spp. counts below 1 log CFU/g were found in 4 antibiotic-free samples (44.44%). The other five samples (55.56%) showed counts between 2.78 and 6.0 log CFU/g, with an average number of 4.41 ± 1.09 log CFU/g. Table 2 shows the *Pseudomonas* spp. distribution, with *P. extremorientalis* and *P. libanensis* being the dominant species.

*Enterobacteriaceae* counts were below the detection limit (<1 log CFU/g) in samples with antibiotic residues. *Enterobacteriaceae* counts below 1 log CFU/g were found in two antibiotic-free samples (22.22%). The other seven samples (77.78%) showed counts between 2.00 and 4.55 log CFU/g, with an average number of 3.49 ± 0.79 log CFU/g. Table 3 shows the species distribution. *Escherichia coli* was the dominant specie, followed by *Serratia liquefaciens*, and *Buttiauxella gavininae*. ESBL-producing *E. coli* was not found in any sample.

*Staphylococci* counts ranged between 1.90 and 4.48 log CFU/g, with an average number of 3.07± 0.84 in antibiotic-free samples, while the counts were 2.21± 0.43 log CFU/g in samples with antibiotic residues. No significant differences (*p* > 0.05) in *staphylococci* counts were found between samples with antibiotic residues and those free of antibiotics. Table 4 shows the distribution of bacteria identified from Mannitol Salt Agar. The predominant specie found in the samples with antibiotic residues were *Aerococcus viridans* followed by *S. epidermidis*, *S. warneri,* and *Kocuria kristinae*. *A. viridans*, a Gram-positive bacterium belonging to the *Aerococcaceae* family, *Lactobacillales* order, was only found in the sample containing 164.1 µg/kg of sulfadiazine. The *Micrococcaceae* found in samples free of antibiotics were *S. equorum* (48.49%), *S. saprophyticus* (21.05%), *Macrococcus caseolyticus* (18.18%), *S. vitulinus* (6.06%), *S. epidermidis* (3.03%), and *S. chromogenes* (3.03%). Methicillin-resistant *S. aureus* was detected in one sample free of antibiotic residues using CHROMID MRSA agar. In addition, *S. epidermidis* and *M. caseolyticus* were isolated from this sample in CHROMID MRSA.

*Enterococci* counts below 1 log CFU/g were found in five antibiotic-free samples (55.56%). The other four samples (44.44%) showed counts between 1.60 and 2.60 log CFU/g, with an average number of 2.17 ± 0.40 log CFU/g. *Enterococci* counts were 1.95 ± 0.65 log CFU/g in samples with antibiotic residues. No significant differences (*p* > 0.05) in *enterococci* counts were found between samples with antibiotic residues and those free of antibiotics. Table 5 shows the species identified from Kanamycin Esculine Azide agar. *E. faecium* was the dominant *enterococci* in antibiotic-free samples (30.77%), followed by *E. hirae* (23.08%). In addition, *Streptococcus gallolyticus*, a Gram-positive bacterium that belongs to the Lancefield group D was isolated. All the *S. gallolyticus* isolates were from one sample. The dominant *enterococci* in samples with antibiotics were *E. faecium*, *E. durans*, and *E. mundtii*. *E.* *faecalis* was only isolated from samples containing antibiotics (14.29%). No vancomycin-resistant *enterococci* were isolated from CHROMID VRE agar.

*Clostridium perfringens* was found in two samples (18.18%) at levels of 1.60–2.30 log CFU/g. One of the samples was free of antibiotics and the other contained 813 µg/kg of doxycycline. Neither *Listeria monocytogenes* nor *Campylobacter* spp. were detected in any sample.

Table 6 shows the antimicrobial resistance phenotype of 24 *staphylococci* and *Macrococcus caseolyticus* isolates from goat meat. It should be noted that 91.67% of the strains were *resistant* to one or more antibiotics, and 45.83% of the strains were multi-resistant (resistance to three or more antibiotic classes. It should be pointed out that one *S. epidermidis* strain showed resistance to 11 antibiotics, while one strain of *S. aureus* showed resistance to 7 antibiotics, both being methicillin-resistant. The highest rates of resistance were observed to tetracycline (70.83% of the isolates). The resistance to doxycycline was observed in 33.33% of the isolates. More than 25% of the isolates showed resistance to sulfadiazine, lincomycine, streptomycin, and erythromycin. More than 10% of the isolates showed resistance to kanamycin, penicillin, and trimethoprim. Resistance to cefoxitin and gentamycin was found in 8.33% of the isolates. Less common was resistance to amikacin, tobramycin, and mupirocin. For antimicrobial classes, the highest resistance corresponded to tetracyclines, followed by lincosamides, folate pathway inhibitors, macrolides, and aminoglycosides. No resistance was observed to fluoroquinolones, nitrofurantoins, glycopeptides, oxazolidones, phenicoles, or ansamycins.

Table 7 shows the antimicrobial resistance phenotype of 14 *enterococci* isolates from goat meat, 8 from samples with presence of antibiotic residues and 6 from samples free of antibiotics. It should be noted that 87.5% of the strains from samples with antibiotic residues were resistant to one or more antibiotics, 75% being multi-resistant, while 50% of the isolates from samples free of antibiotics were resistant to one or more antibiotics, and 33.33% were multi-resistant. Of the 14 isolates, 50% showed resistance to tetracycline, 42.86% to nitrofurantoin, 35.71% to enrofloxacin, 21.43% to ciprofloxacin, and 14.29% to norfloxacin. For antimicrobial classes, the highest resistance corresponded to tetracyclines, followed by nitrofurantoins and fluoroquinolones. No resistance was observed to glycopeptides, phenicoles, or oxazolidones.

## 4. Discussion

Similar mesophile counts have been reported by other authors in fresh goat meat [1,32]. By contrast, other authors have reported higher mesophile counts [10]. The microbial contamination of meat depends on the hygienic conditions, the handling, and the conditions of storage (time and temperature) [1]. We did not find significant differences (*p* > 0.05) in mesophile counts between the samples with antibiotic residues and those free of antibiotics. However, significant differences (*p* < 0.05) in mesophiles were found between the sample with doxycycline at levels of 813 µg/kg and the sample with sulfadiazine at levels of 164.3 µg/kg. Information comparing microbial load or dominant bacteria in meat with and without antibiotics is not available in the bibliography. In the present work, the predominant bacteria isolated from Plate Count Agar in samples with antibiotics differed from those observed in antibiotic-free samples. Lower diversity was observed in the samples containing antibiotics.

The microbiological quality of meat depends on several factors, such as the animal conditions, the spread of contamination during slaughter and further processing, and the storage conditions [27]. These factors could explain the differences in the dominant bacteria reported by other authors [1]. In the present study, *Pseudomonas* spp. were only isolated from antibiotic-free samples. *Pseudomonas* spp. are important spoilage bacteria, and their spoilage capacity could be specie-dependent [6]. On the other hand, some species can be human pathogens, such as *P. aeruginosa* [6]. We did not isolate *P. aeruginosa* in any meat sample. However, Bantawa et al. [10] reported a high prevalence of *P. aeruginosa* (33.33%) in fresh goat meat.

*Enterobacteriaceae* counts below 1 log CFU/g were found in all the samples containing antibiotics. In contrast, the antibiotic-free samples showed counts between <1 and 4.55 log CFU/g. *Escherichia coli* was the dominant specie, followed by *Serratia liquefaciens* and *Buttiauxella gavininae*. In contrast, Carrizosa et al. [2] reported that the dominant *Enterobacteriaceae* isolated from fresh goat meat was *S. liquefaciens*. Moreover, Carrizosa et al. [2] did not isolate *E. coli* or *B. gavininae* from goat meat. The main source of the microbiological contamination of carcasses along the slaughter line is of fecal origin; therefore, *Enterobacteriaceae* can be used as an indicator of the hygienic status of the slaughter [14]. Although we did not isolate any extended-spectrum β-lactamase (ESBL)-producing *E. coli*, its evaluation is important, since its presence in goat meat has been reported by other authors [19].

*Clostridium* spp. has also been detected in fresh goat meat by other authors [32]. We detected C. *perfringens* in one sample containing antibiotics (50%), and another free of antibiotics (11.11%). *C. perfringens* is an inhabitant of the intestinal tract of animals; thus, the meat contamination could be related to fecal contamination [33]. *C. perfringens* outbreaks have been associated with the consumption of meat prepared in very large quantities and inadequately cooked, mainly roast beef and mutton kebab dishes [34]. Measures should be taken to avoid goat meat contamination with *C. perfringens*. On other hand, special care should be taken in goat meat cooking and handling if it is roasted and maintained at inadequate temperatures before consumption.

In the present study, *L. monocytogenes* was not detected. Other authors have reported a low prevalence of *Listeria* spp. in goat meat (1.78%) [35]. However, other authors found a higher prevalence of *L. monocytogenes* in goat meat (33.33%) [13].

As in the study by Kim et al. [13], *Campylobacter* spp. was not detected in any goat meat sample. In contrast, Lazou et al. [36] reported a prevalence of 30.2% in goat meat from Greece.

Similar counts of *staphylococci* have been reported by Cherroud et al. [37] in fresh goat meat. In the current study, no significant effect (*p* > 0.05) of the presence of antibiotics on *staphylococci* counts was observed when compared to the samples free of antibiotics. However, the species found were different. The main *staphylococci* found in goat meat free of antibiotics were *S. equorum* followed by *S. saprophyticus*. By contrast, Carrizosa et al. [2] reported that the main *Staphylococcus* spp. present was *S. saprophyticus*. These authors also isolated *M. caseolyticus* from goat meat. The genera *Macrococcus* belongs to the family *Staphylococcaceae* and is closely related to the genera *Staphylococcus* [38]. Other authors have reported a high prevalence of *S aureus* (70%) in fresh goat meat [10]. We isolated *Aerococcus viridans* from one meat sample containing 164.1 µg/kg of sulfadiazine. This bacterium is considered an opportunistic pathogen [39]. *A. viridans* has been isolated from goat milk and milk products [40]. As far as we know, the presence of *A. viridans* in goat meat has not been previously reported, and it should be noted that this bacterium was only isolated from one sample containing antibiotics. Some studies indicate that *A. viridans* is highly resistant to antimicrobials [39]. As *A. viridans* might be a potential pathogen with high antimicrobial resistance, further studies are needed to know the extent of its presence in goat meat and the role of antibiotic residues in selecting this microorganism.

It should be noted that the *staphylococci* isolated from the samples containing 813 µg/kg doxycycline were *S. epidermidis* and *S. warneri.* These isolates showed resistance to mupirocin and tetracycline, respectively. It should be pointed out that mupirocin is an important antibiotic for the treatment of infections in humans, and it has been categorized as “Category A: antimicrobial to avoid” in animals; moreover, its use is not authorized in veterinary medicine in the EU [41].

Most of the *staphylococci* and *M. caseolyticus* that were multidrug-resistant were isolated from one sample free of antibiotics. We detected methicillin-resistant *S. aureus* as well as *S. epidermidis* and *M. caseolyticus* in that sample using CHROMID MRSA agar. The *S. aureus* isolate was multidrug-resistant, being resistant to tetracycline, cefoxitin, penicillin, benzilpenicillin, kanamycin, streptomycin, and erythromycin. Sergelidis et al. [21] reported that all isolates of *S. aureus* from small ruminants were resistant to at least one antibiotic, and 59.3% were multidrug-resistant. These authors observed high resistance rates to penicillin (100%), tetracycline (74%), clindamycin (59.3%), and erythromycin (51.9%), whereas resistance to cefoxitin was observed in 22.2% of the isolates. Tefera et al. [42] also observed that *S aureus* isolated from small ruminant carcasses presented high resistance rates to penicillin, cefoxitin, and erythromycin. De Miranda et al. [43] also reported that *S. aureus* isolated from goat meat exhibited resistance to penicillin and tetracycline and to other antimicrobials. We observed that *S epidermidis* isolated from MRSA presented resistance to tetracycline, doxycycline, cefoxitin, penicillin, benzilpenicillin, kanamycin, gentamicin, streptomycin. Tobramycin, and sulfadiazine, while *M. caseolyticus* isolated from MRSA medium exhibited resistance to tetracycline, doxycycline, penicillin, kanamycin, lincomycine, and gentamicin. The strains of *S. epidermidis* and *M. caseolyticus* isolated from the same sample but from MSA showed the following antimicrobial phenotypes: tetracycline–streptomycin–erythromycin and tetracycline–streptomycin–sulfadiazine–lincomycine, respectively. Thus, the strains isolated from MSA showed lower antimicrobial resistance than those isolated from the chromogenic agar. Other strains isolated from this sample in MSA were *S. equorum* (antimicrobial phenotype: tetracycline–erythromycin–lincomycine–trimethoprim) and *S. saprophyticus* (antimicrobial phenotype: tetracycline–doxycycline). *S. equorum* was isolated from another sample purchased in a different shop but from the same brand, showing the same antimicrobial phenotype (tetracycline–erythromycin–lincomycine–trimethoprim). Moreover, although these samples were purchased in different shops than the sample that presented doxycycline residues, their origin was the same brand.

We did not identify any *staphylococci* from the sample containing 164.1 µg/kg sulfadiazine. However, we isolated *S. equorum, S. saprophyticus,* and *M. caseolyticus* from another sample of the same brand. These strains showed the following antimicrobial resistance phenotypes: tetracycline–doxycycline–streptomycin–lincomycine–trimethoprim, tetracycline–doxycycline–streptomycin, and tetracycline, respectively.

*S. equorum*, *S. saprophyticus*, *S. vitulinus*, and *M. caseolyticus* were also isolated from other antibiotic free samples from other brands. These strains showed no resistance or resistance to one or two different antimicrobial classes. None of these isolates showed multidrug-resistance. In contrast, all the multidrug-resistance isolates presented the same brand origin as the samples containing antibiotic residues. These results suggest that the farm practices and environment could affect the antimicrobial resistance pattern. Our results show that all the *staphylococci* and *M. caseolyticus* isolated were susceptible to fluoroquinolones, oxazolidones, phenicols, and fucsidic acid, among others.

In the present work some of the isolates of *M. caseolyticus* showed multidrug-resistance. This fact is of special interest, since some authors have pointed out its potential for disseminating antimicrobial resistance [44]. Other authors have also reported that *Macrococcus* spp. isolated from food-producing animals and meat exhibited resistance to tetracycline, penicillin, streptomycin, and gentamicin [38].

Coagulase-negative *staphylococci* (*S. chromogenes*, *S. epidermidis, S. equorum*, and *S*. *saprophyticus*) could be a reservoir of clinically relevant resistance genes that could be transferred to *S. aureus* isolates [45]. We observed that most of the coagulase-negative *staphylococci* showed resistance to at least one tested antimicrobial agent.

The use of antibiotics in food-producing animals can act as a selective pressure for resistant bacteria [45]. Tetracyclines, sulphonamides, quinolones, and β-lactams are frequently used in the treatment of food-producing animals [46]. The resistance to tetracyclines, sulphonamides, and β-lactams observed in the present work could be related to the antimicrobial classes often used in farm animal infection treatments. On other hand, we observed resistance to antibiotics that are categorized as “Category C: caution” for use in animals such as kanamycin, streptomycin, erythromycin, amikacin, tobramycin, gentamycin, and lincomycine [41].

We did not find significant differences (p > 0.05) in *enterococci* counts between samples with antibiotic residues and those free of antibiotics. However, differences between the species found were observed. *E. faecium* was the dominant *enterococci* in both antibiotic-free and the samples with antibiotic residues. *E. faecalis*, and *E. durans* were only isolated from samples containing antibiotics. As in Cherroud et al. [37], we observed that *E. faecium* was the dominant *Enterococcus* spp. in goat meat. In contrast, Carrizosa et al. [2] reported that *E. faecalis* was the dominant *enterococci* member in fresh goat meat. Carrizosa et al. [2] also isolated *E. durans* and *E. hirae* in fresh goat meat. *Enterococci* are used in fresh meat as an indicator of fecal contamination. In consequence, low counts indicate good hygiene processing conditions [47].

Few studies include both the analysis of residues in meat and the antibiotic resistance [48]. We observed that 87.5% of the *enterecocci* strains from samples with antibiotic residues were resistant to one or more antibiotics, with 75% being multi-resistant, while 50% of the isolates from samples free of antibiotics were resistant to one or more antibiotics, and 33.33% were multi-resistant. All the multi-resistant *enterococci* were isolated from the same brand as the samples containing antibiotics. For antimicrobial classes, the highest resistance corresponded to tetracyclines, followed by nitrofurantoins and fluoroquinolones. It should be noted that some *enterococci* isolates showed resistance to high levels of tetracycline, with MIC of 32 and 256 µg/mL It has been reported that tetracycline-resistant *enterococci* of animal origin are often resistant to other antimicrobial agents [49]. Rodrigues et al. [50] reported that *E. faecalis* and *E. mundtii* isolated from animals showed resistance to tetracycline and norfloxacin. As in the present work, these authors reported that all the *enterococci* isolates were susceptible to chloramphenicol and vancomycin. However, the mentioned authors did not observe any resistance to ampicillin, ciprofloxacin, levofloxacin, and nitrofurantoin. In the present work, five of seven *E. faecium* isolates shown resistance to fluoroquinolones. In general, the minimum inhibitory concentration (MIC) for enrofloxacin and ciprofloxacin was higher in the enterococci isolated form samples containing antibiotics, with levels of 4–6 µg/mL and 2–3 µg/mL, respectively. It should be noted that fluoroquinolones are important antibiotics for the treatment of infections in humans and they have been categorized as “highest priority critically important antimicrobials”, and their use in animal should be restricted [32]. We also observed resistance to tigecycline in two isolates form *E. mundtii* and one isolate of *E. faecium*. This finding is relevant since tigecycline is categorized in “Category A: antimicrobial to avoid” for animals; moreover, its use is not authorized in veterinary medicine in the EU [41].

In the present work, most of the multi-resistant bacteria were isolated from the same brands as the samples containing antibiotics. These results suggest that farm practices and environment could play an important role in antimicrobial resistance. Other authors have reported that farm conditions, farm environment, farm hygiene, and contact with humans could affect the presence of resistant bacteria in food-producing animals, and they should be considered to control antimicrobial resistance dissemination [51,52,53,54,55]

Special care should be taken in the slaughter process to avoid the fecal contamination of goat meat, since *enterococci* are normally present in the intestinal tract and could be multi-resistant bacteria [46]. The cross-contamination during the slaughter process may be a food safety risk [56] as it might be enhancing the already serious problem of antimicrobial resistance dissemination.

## 5. Conclusions

Our results suggest that the presence of sulfadiazine or doxycycline residues can affect the goat meat microbiota, and in some cases enhances the antimicrobial resistance of bacteria found in goat meat. The presence of antibiotic residues could increase the antimicrobial resistance of *enterococci* in fresh goat meat. Since most of the multi-resistant bacteria were isolated from the same brands as the samples in which residues were detected, it seems that farm practices and environment could affect the antimicrobial resistance rates. The presence of multidrug-resistant *S. aureus*, and especially MRSA, in goat meat, could be considered a potential threat and should be monitored.

Further studies are needed to evaluate the extent of *A. viridans* in fresh goat meat and the role of antibiotic residues in selecting this microorganism.

*C. perfringens* was isolated in 18.18% of the samples, representing a risk for human health. Measures should be taken to avoid goat meat contamination with *C. perfringens*. On other hand, special care should be taken in goat meat cooking and handling if it is roasted and maintained at inadequate temperatures before consumption.

## Figures and Tables

**Table 1:** Bacteria identified in fresh goat meat from Plate Count Agar. Samples with the presence of antibiotic residues (P) and free of antibiotic residues (N).

Antibiotic Residues	Microbial Group or Genera	Percentage(%)	Species	Percentage (%)
Yes (P)	Lactic acid bacteria	58.33	*Lactobacillus* spp. *Carnobacterium divergens* *Carnobacterium maltaromaticum*	25.00 25.00 8.33
	*Brocchotrix thermosphacta*	16.67	*B. thermosphacta*	16.67
	Other (Gram negative Bacteria)	25.00	*Microbacterium oxydans* *Microbacterium esteraromaticus Psychrobacter maritimus*	8.33 8.33 8.33
	Total	100		100
No (N)	*Brocchotrix thermosphacta*	35.85	*B. thermosphacta*	35.85
	*Pseudomonas spp*	26.42	*P. fragi* *P. lundensis* *P. extremorientalis* *P. brenneri* *P. chlororaphis*	15.09 3.77 3.77 1.89 1.89
	*Micrococcaceae*	13.21	*Staphylococcus vitulinus* *Macrococcus caseolyticus* *Staphylococcus simulans* *Staphylococcus equorum* *Kocuria rhizophila*	3.77 3.77 1.89 1.89 1.89
	*Enterobacteriaceae*	5.66	*Buttiauxella gaviniae* *Escherichia coli*	3.77 1.89
	*Enterococci*	3.77	*Enterococcus faecalis* *Enterococcus hirae*	1.89 1.89
	Other (Gram negative Bacteria)	15.09	*Sphingobacterium faecium* *Acinetobacter johnsonii* *Acinetobacter lwoffii* *Chryseobacterium scophtalnum* *Brevundimonas diminuta*	5.66 3.77 1.89 1.89 1.89
	Total	100	5	100

**Table 2:** Percentage of *Pseudomonas* spp. isolated from chromogenic agar in fresh goat meat free of antibiotic residues.

Specie	Percentage (%)
*Pseudomonas extremorientalis*	42.87
*Pseudomonas libanensis*	19.05
*Pseudomonas fluorescens*	9.52
*Pseudomonas tolaasii*	9.52
*Pseudomonas fragi*	4.76
*Pseudomonas fulva*	4.76
*Pseudomonas synxantha*	4.76
*Pseudomonas veronii*	4.76
Total *Pseudomonas* spp.	100

**Table 3:** Percentage of *Enterobacteriacceae* isolates identified in fresh goat meat free of antibiotic residues.

Specie	Percentage (%)
*Escherichia coli*	50
*Serratia liquefaciens*	25
*Buttiauxella gavininae*	25
Total *Enterobacteriacceae*	100

**Table 4:** Percentage of isolates identified in fresh goat meat from Mannitol Salta Agar. Samples with the presence of antibiotic residues (P) and free of antibiotic residues (N).

Antibiotic Residues	Specie	Percentage (%)
Yes (P)	*Aerococcus viridans*	50.00
	*Staphylococcus epidermidis*	20.00
	*Staphylococcus warneri*	20.00
	*Kocuria kristinae*	10.00
	Total	100.00
No (N)	*Staphylococcus equorum*	48.49
	*Staphylococcus saprophyticus*	21.21
	*Macrococcus caseolyticus*	18.18
	*Staphylococcus vitulinus*	6.06
	*Staphylococcus epidermidis*	3.03
	*Staphylococcus chromogenes*	3.03
	Total	100

**Table 5:** Percentage of isolates identified in fresh goat meat from Kanamycin Esculin Azide agar. Samples with the presence of antibiotic residues (P) and free of antibiotic residues (N).

Antibiotic Residues	Specie	Percentage (%)
Yes (P)	*E. faecium*	28.57
	*E. durans*	28.57
	*E. mundtii*	28.57
	*E. faecalis*	14.29
	Total	100
No (N)	*E. faecium*	30.77
	*E. hirae*	23.08
	*Streptococcus gallolyticus*	38.46
	*Vagococcus lutrae*	7.69
	Total	100

**Table 6:** Antimicrobial resistance phenotype of *staphylococci* and *Macrococcus caseolyticus* isolated from goat meat. Samples with the presence of antibiotic residues (P) and free of antibiotic residues (N.).

Antibiotic Residues	Specie (Number of Isolates)	Antibiotic Resistance Phenotype ^1^ (Number of Isolates)
Yes (P)	*Staphylococcus epidermidis* (1)	PUM (1) ^2^
	*Staphylococcus warneri* (1)	TE (1) ^2^
No (N)	*Staphylococcus aureus* (1)	TE- FOX-P-PNG-K-S-ERY (1) ^3,4^
	*Staphylococcus chromogenes1*	P (1) ^2,4^
	*Staphylococcus epidermidis* (2)	TE-DO-FOX-P-K-AK-CN-S-TOB-ERY-SUZ- (1) ^3,4^
		TE-S-ERY(1) ^2,4^
	*Staphylococcus equorum* (5)	ERY (1) ^1^
		TE-ERY-MY-W (2) ^2,4^
		ERY-MY (1) ^2^
		TE-DO-S-MY-W (1) ^2,4^
	*Staphylococcus saprophyticus* (5)	TE-DO-MY (1) ^2^
		susceptible to all antibiotics tested (1) ^2^
		TE-DO (2) ^2^
		TE-DO-S (1) ^2,4^
	*Staphylococcus vitulinus* (2)	TE-DO-MY (1) ^2^
		TE (1) ^2^
	*Macrococcus caseolyticus* (6)	susceptible to all antibiotics tested (1) ^2^
		TE-MY (1) ^2^
		S (1) ^2,4^
		TE-S-SUZ-MY (1) ^2,4^
		TE-DO-P-K-MY-CN (1) ^3,4^
		TE (1) ^2^

^1^ PUM: Mupirocin, TE: tetracycline, FOX: Cefoxitin, P: Penicillin, PNG: Benzilpenicillin, K: Kanamycin, S: Streptomycin, ERY: erythromycin, P, penicillin, DO: Doxycycline, AK: Amikacin, CN: Gentamycin, TOB: tobramycin, SUZ: Sulfadiazine, MY: Lincomycine, W: Trimethoprim. ^2^ Strain isolated from Mannitol Salt Agar (MSA). ^3^ Strain isolated from MRSA. ^4^ Strain isolated from samples of the same brand that those containing antibiotics.

**Table 7:** Antimicrobial resistance phenotype of *enterococci* isolated from goat meat. Samples with the presence of antibiotic residues (P) and free of antibiotic residues (N).

Antibiotic Residues	Specie (Number of Isolates)	Antibiotic Resistance Phenotype ^1^ (Number of Isolates)	MIC ^3^ TE (µg/mL)	MIC ENRO/CIP (µg/mL)
Yes (P)	*E. durans* (1)	TE-DO-MH-F (1)	256	NI/NI
	*E. faecalis* (1)	TE (1)	32	NI/NI
	*E. faecium* (4)	TE- MH- F-Enro (1)	6	1.5/NI
		TE-DO-MH-ENRO-CIP-NOR-F (1)	12	4/2
		TE-DO-MH-ENRO-CIP-NOR-F (1)	8	6/3
		Sensivity	NI	NI/NI
	*E. mundtii* (2)	MH-TGC (1)	NI	1/0.25
		F-TEC-TGC (1)		NI/NI
No (N)	*E. faecium* (3)	susceptible to all antibiotics tested (1)	NI	NI/NI
		TE-DO-MH-ENR-CIP-AMP-IMP-F-TGC (1) ^2^	32	1.5/1.5
		TE-DO-MH-ENR-CIP-IMP-F (1) ^2^	24	2/1.5
	*E. hirae* (3)	susceptible to all antibiotics tested (1) ^2^	NI	NI/NI
		MH (1)	NI	NI/NI
		susceptible to all antibiotics tested (1) ^2^	NI	NI/NI

^1^ TE: tetracycline; DO: doxycycline; MH: minocycline; F: nitrofurantoin; ENR: enrofloxacin; CIP: ciprofloxacin; NOR: norfloxacin; TGC: tigecycline; TEC: teicoplanin; AMP: ampicillin; IMP: imipenem. ^2^ Strain isolated from samples of the same brand that those containing antibiotics. ^3^ minimum inhibitory concentration (MIC).

## Data Availability

The date are available from the corresponding author.
